# Political news on Instagram: influencer versus traditional magazine and the role of their expertise in consumers’ credibility perceptions and news engagement

**DOI:** 10.3389/fpsyg.2023.1257994

**Published:** 2023-12-06

**Authors:** Daniel Zimmermann, Asina Klee, Kai Kaspar

**Affiliations:** Department of Psychology, University of Cologne, Cologne, Germany

**Keywords:** social networking sites, political news, Instagram, expertise, credibility, news engagement

## Abstract

**Introduction:**

Social networking sites (SNS) are increasingly used by consumers to read and share political news. In this context, Instagram plays an important role due to its prevalence and visual characteristics. However, previous research has highlighted that consumers fail to identify the source of online news, though source characteristics were shown to be vital for news credibility perceptions. Nevertheless, research on whether and which source characteristics have an influence on Instagram consumers’ credibility perceptions and news engagement intentions are lacking. The present study addresses this empirical gap by investigating potential effects of source expertise and source type on source credibility, message credibility, news engagement intentions, and personal involvement regarding political news on Instagram.

**Method:**

We randomly presented participants with political news posts from one of four sources, either the Instagram representation of a fictional news magazine or influencer with or without political expertise. Participants assessed the perceived credibility of the source and the news, their news engagement intentions, and personal involvement.

**Results:**

We analyzed data from 416 participants. Results showed significant main effects of source expertise on each of the dependent variables. Those were shown to be indirect effects through personal involvement. There were hardly any effects of source type.

**Discussion:**

These results provide new insights into the role of source expertise on credibility perceptions and news engagement intentions, and provide insights into the comparison between influencers and Instagram representations of traditional news magazines. Theoretical implications for future research and practical implications for content creators, users, and SNS platforms are discussed.

## 1 Introduction

Despite being originally developed for social interaction, social networking sites (SNS) such as Instagram, YouTube, and Facebook contain a large amount of political communication and information that affects consumers’ political opinions and perspectives (Johnson and Kaye, 2014). SNS have been shown to affect news engagement, i.e., reading and sharing news with others (Lee and Ma, 2012; Ma et al., 2014). Furthermore, the consumption of news on SNS can affect political knowledge and political participation (Park and Kaye, 2018). In Germany, for example, SNS are already used as primary news sources with similar frequency as online news magazines (Hölig et al., 2020). However, the immense amount of accessible information online may overwhelm consumers with the selection of information and the assessment of its credibility, which in turn further increases the amount of online misinformation (Talwar et al., 2019; Laato et al., 2020; Apuke and Omar, 2021). One key problem in this regard is the lack of professional gatekeepers who verify, arrange, and filter online information before it gets published (e.g., Metzger, 2007; Johnson and Kaye, 2014), as anyone can spread and post news on SNS. Therefore, a check of the source and the producer’s qualifications are proposed as a central factor for the evaluation of credibility of online information (Metzger, 2007), which is a vital criterion related to the behavior and attitudes of consumers regarding a message (Wathen and Burkell, 2002; Rieh and Danielson, 2007).

One of the most important sources of (political) news thereby are SNS representations of traditional news media (e.g., magazine, newspaper, TV news, radio). Consequently, traditional news media use SNS as a platform to distribute their news and (sometimes) redirect consumers from SNS to their own websites by providing links to them (Hille and Bakker, 2013). Indeed, traditional newspapers’ SNS representations were found to have a strong effect on news distribution and consumer engagement (Welbers and Opgenhaffen, 2018). The SNS platform Instagram plays a special role in this context nowadays, as it is used especially by young people to share messages, show their own opinions, and draw the attention of other peers due to the simplicity of sharing posts and reaching their own peer group (Wang, 2021). Moreover, and analogously, Vázquez-Herrero et al. (2019) showed that the number of Instagram profiles of traditional news outlets is steadily increasing and adapting their messages to the specific format of the platform and to consumers’ preferences (Vázquez-Herrero et al., 2019). Nevertheless, news consumption has so far been studied mainly for platforms such as online blogs (e.g., Yuan et al., 2019) and Facebook (e.g., Meinert and Krämer, 2020), while there is little research, especially experimental studies, on news consumption on Instagram. The present study closes this gap by examining news perceptions and engagement intentions in the context of Instagram.

Importantly, not only traditional news media use SNS to distribute news. So-called *political influencers* also gain an increasingly important role as a new generation of gatekeepers on SNS, as they report and share political information increasingly often and use their social reach to engage with politics and current affairs (Bause, 2021; Fischer et al., 2022). In fact, political influencers were shown to have a positive effect on consumers’ response rate and the political agenda (Curiel, 2020) as well as on political interest (Schmuck et al., 2022). Though, critically, not only influencers with topic-related expertise distribute political information, but also those who have not (Trepte and Scherer, 2010). Therefore, the source’s expertise, defined as “the level of skill or knowledge of the communicator individuals perceive” (Yuan et al., 2019, p. 272), is one of the key characteristics that influences whether people trust a producer’s message or not (Pavlíčková, 2013). Expertise is a central heuristic that is used to make judgments about the credibility of a message’s source (Metzger et al., 2010; Sterrett et al., 2019; Meinert and Krämer, 2022). This source credibility is in turn an important factor in assessing the credibility of the message (Kang et al., 2011) and engaging with it (Keib and Wojdynski, 2019). The assessment of source credibility through its expertise might be particularly present on SNS where news is often consumed as snacks, i.e., rather incidentally, occasionally, quickly, and only in parts (Bergström and Jervelycke Belfrage, 2018; Keib et al., 2021). This forces consumers to assess the credibility of a news message in a short time by using heuristic ways of information processing (Flanagin and Metzger, 2000; Metzger et al., 2010), indicating that the perceived expertise of a source is a crucial factor in the credibility assessment of news messages on SNS.

Critically, though, consumers seem to have difficulties with recognizing the source and utilizing it to evaluate news on SNS. Pearson (2021) defines the problem of “*source blindness”* (p. 1182) on SNS as design features reducing the attention to the source, because they are the same for every source, meaning that the specific contextual and structural properties of SNS can lead to general source blindness. This suggests that the source and its characteristics, such as expertise, may not be considered appropriately when evaluating news messages on SNS. However, with respect to political news on SNS, it is still an empirically open question whether and, if so, to what extent consumers consider the source when evaluating their news messages. The present study tackles this question by examining the potential effects of the source type of political news distributed via SNS (i.e., SNS representation of traditional news magazine vs. influencer) and the topic-related expertise of the source (i.e., with political expertise vs. without political expertise) on consumers’ perception of source credibility and message credibility, as well as on their engagement intentions with the news.

## 2 Literature review

In the following, based on theory and empirical evidence, we outline the role of source expertise and source type for perceived source credibility, message credibility, and news engagement intentions. Possible mediating roles of credibility and personal involvement are also outlined. The hypotheses of the present study are then derived accordingly.

### 2.1 The credibility of source and news message

In general, credibility is an essential criterion related to the behavior and attitudes of consumers regarding a message and its source, which has been examined in different disciplines like information science, communication, and psychology (Wathen and Burkell, 2002; Rieh and Danielson, 2007). A differentiation is thereby drawn between source credibility and message credibility. While message credibility is defined as “an individual’s judgment of the veracity of the content of communication” (Appelman and Sundar, 2016, p. 63), source credibility is referred to as the credibility of the message’s source (Pornpitakpan, 2004). In general, message credibility results from the interaction of characteristics of the source, characteristics of the message itself (e.g., content, plausibility, quality), and characteristics of the receiver (e.g., social background, beliefs, knowledge) (Wathen and Burkell, 2002). Accordingly, source credibility and message credibility are shown to be positively connected. The message is perceived as more credible if the source itself is perceived as credible as well (Kang et al., 2011), and a source with high credibility was found to be more persuasive and therefore more likely to influence a person’s attitudes and behavior (for a review, see Pornpitakpan, 2004). Furthermore, Keib and Wojdynski (2019) showed that consumers of TV news on Facebook were more willing to engage with this news (i.e., sharing, clicking through, and liking the news), when the news was perceived as credible. Thus, source credibility and message credibility seem to be important factors for consumers’ news engagement intentions in the context of SNS.

### 2.2 The role of source expertise in assessing credibility and inducing news engagement

The perceived expertise of a source plays an important role in the evaluation of source credibility and message credibility. On the one hand, according to the Source Credibility Theory (*cf.* Lowry et al., 2014), expertise is one of the most important factors influencing the persuasiveness and perceived usefulness of a message. On the other hand, expertise also was identified as one of the underlining concepts in the evaluation of the source credibility (Ohanian, 1990). The role of the expertise of news sources on SNS for their credibility and their content has mainly been examined in the context of influencer marketing (e.g., Xiao et al., 2018; Lou and Yuan, 2019; Lee and Kim, 2020). For example, the more a source was perceived as knowledgeable or competent – e.g., through a brand name in the context of marketing – the higher the credibility of the source and, ultimately, the message were rated online (Choi and Stvilia, 2015; Jenkins et al., 2020). In the context of political news, the source’s expertise is essential for the assessment of the credibility of SNS in general (for an overview, see Metzger and Flanagin, 2015). So, for example, Eastin (2001) showed that online health messages were perceived as more credible when the source of the message had high expertise and when it was knowledgeable about the content. Yuan et al. (2019) also found positive effects of the author’s expertise on perceived message credibility in online blog posts. Meinert and Krämer (2020) showed that the expertise of politicians positively influenced the perceived credibility of their political statements on Facebook. These results from related research areas suggest that source expertise plays an important role in assessing source credibility and message credibility. Though, the role of perceived expertise of sources on SNS in assessing their credibility and the credibility of the messages they share has not, to our knowledge, been studied in the context of political news on SNS. This study fills this gap by examining the effect of the source’s political expertise on the evaluation of perceived source credibility and perceived news message credibility. Additionally, due to previously found connections between message credibility and news engagement intentions (Keib and Wojdynski, 2019) we also explored a potential effect of source expertise on consumers’ news engagement intentions:

*H1*: In the context of Instagram, there is an effect of source expertise (with political expertise vs. without political expertise) on perceived source credibility (H1a), perceived message credibility (H1b), and consumers’ news engagement intentions (H1c).

### 2.3 The role of source type in assessing credibility and inducing news engagement

In addition to source expertise, there are indications that the source type may affect the perception of source credibility and, in turn, news engagement intentions. Traditional media including magazines, newspapers, TV, radio, and their online counterparts, is still perceived as more credible as a source compared to original SNS sources (Johnson and Kaye, 2014), and traditional media is more trusted than SNS, despite the prevalence of the latter (Li and Zhang, 2018). Accordingly, media credibility was only positively associated with the role conception of professional journalists, but not with the conception of citizen journalists, which emphasizes the importance of professionalism in journalism to enhance credibility (Nah and Chung, 2012). Furthermore, adolescents reported to perceive traditional news as more objective, credible, serious, and professional compared to political YouTube videos of influencers, whereas the latter were perceived as more subjective and manipulative, thus reflecting a higher trust in more traditional news media (Zimmermann et al., 2020). In addition, news articles shared by news organizations were perceived as more credible than the same articles shared by their SNS friends (Tandoc, 2019). Besalú and Pont-Sorribes (2021) also found that news presented in a traditional news format (digital newspaper and digital television) was perceived as more credible than the same news presented in a SNS format (Facebook and WhatsApp). Importantly, this also led to higher willingness to share the news presented in the more traditional format. So, previous studies suggest that the evaluation of source credibility may depend on the type of source sharing the news, namely more traditional news formats are still perceived as more credible than news distributed via SNS. Whether this also applies to representations of traditional news media on SNS remains to be shown. The present study thus tackled this issue by examining the effect of source type (SNS representation of traditional news magazine vs. influencer) on perceived source credibility, perceived message credibility, and news engagement intention in the context of Instagram:

*H2*: In the context of Instagram, there is an effect of source type (SNS representation of traditional news magazine vs. influencer) on perceived source credibility (H2a), perceived message credibility (H2b), and consumers’ news engagement intentions (H2c).

### 2.4 The role of source expertise and source type in inducing personal involvement

Because individual people perceive information and its credibility differently, for example, because of different motivations (Armstrong and McAdams, 2009), the attributes of the receiver also are a crucial factor for the assessment of credibility (Wathen and Burkell, 2002). In the context of information processing and perceived credibility, dual-processing theories such as the Elaboration Likelihood Model (Petty and Cacioppo, 1986) and the Heuristic Systematic Model (Chaiken, 1980; Todorov et al., 2002) especially focus on the person’s involvement. The models propose that people who are capable and motivated to analyze content more thoroughly (i.e., focus on arguments, quality, and content) do so because they believe that their opinion has significant consequences (Chaiken, 1980; Petty and Cacioppo, 1986; Metzger, 2007). Low involvement, on the other hand, causes people to evaluate content less systematically, i.e., focus more on the likeability or reputation of the source (Chaiken, 1980; Petty and Cacioppo, 1986; Metzger, 2007; Kang et al., 2011; Xiao et al., 2018).

Importantly, previous research found positive associations between the perceived credibility of the source of information, the perceived credibility of the information itself, the person’s involvement, and behavioral intentions (i.e., purchase decisions, electronic word of mouth) mainly in the context of marketing (e.g., Kautsar et al., 2012; Xiao et al., 2018). However, previous studies have rather focused on the moderating role of involvement by comparing the effects of source characteristics such as expertise (Wiedmann and Von Mettenheim, 2020) or source type (Chiu and Ho, 2023) on the effectiveness of influencer marketing for different levels of product involvement. The questions of whether consumer’s involvement itself is also influenced by source characteristics such as source expertise and source type have been neglected. Ziegele et al. (2017) already showed that news context factors such as news value affect consumer’s involvement in a news article. Thus, we transfer this account to news on Instagram by examining effects of source expertise and source type on consumers’ involvement in political news. We extended H1 and H2 accordingly with the following additional expectations:

*H1d*: In the context of Instagram, there is an effect of source expertise (with political expertise vs. without political expertise) on consumers’ involvement in news messages.

*H2d*: In the context of Instagram, there is an effect of source type (SNS representation of traditional news magazine vs. influencer) on consumers’ involvement in news messages.

### 2.5 Potential interactions between source expertise and source type

As outlined above, there are good reasons to assume that source expertise and source type each have an effect in the context news messages distributed via SNS. In addition to these main effects, the impact of expertise may depend on the source type. Especially in the case of influencers, the role of expertise in assessing credibility could be even more incisive, as not only influencers who know the specific fields share information, but also those who have little knowledge about it (Trepte and Scherer, 2010). In contrast, more traditional media are rather seen as credible and professional *per se* (Zimmermann et al., 2020). Thus, perceived source credibility, perceived message credibility, consumers’ news engagement intentions, and their involvement in news messages may be more dependent on expertise as a heuristic criterion in case of SNS influencers compared to the SNS representations of more traditional news outlets. Thus, we examined a possible interaction effect between source expertise and source type on those dependent variables:

*H3*: In the context of Instagram, there is an interaction effect between source expertise and source type on perceived source credibility (H3a), perceived message credibility (H3b), consumers’ news engagement intentions (H3c), and consumers’ involvement in news messages (H3d).

### 2.6 The mediating role of perceived credibility and personal involvement on news engagement

Finally, credibility and involvement also seem to potentially mediate effects between news context factors and news engagement intentions. For example, Curry and Stroud (2021) found that the perception of the credibility of a news organization mediated the effect of journalistic transparency on news engagement intentions. Jiang et al. (2020) showed partial mediation effects of message credibility for the effect of news’ framing on consumer’s viewing behavior regarding health messages headlines. At last, indirect effects of the news value on the willingness to reply to a comment to a news article via the cognitive involvement with that news article could be found (Ziegele et al., 2017). Thus, we hypothesize that if there is an effect of source expertise and source type on news engagement intentions, there are indirect effects via perceived source credibility, perceived message credibility, and personal involvement (H4).:

*H4*: If there are effects of source expertise and source type on news engagement intentions, these are indirect effects via perceived source credibility, perceived message credibility, and consumers’ involvement.

**Figure 1:**
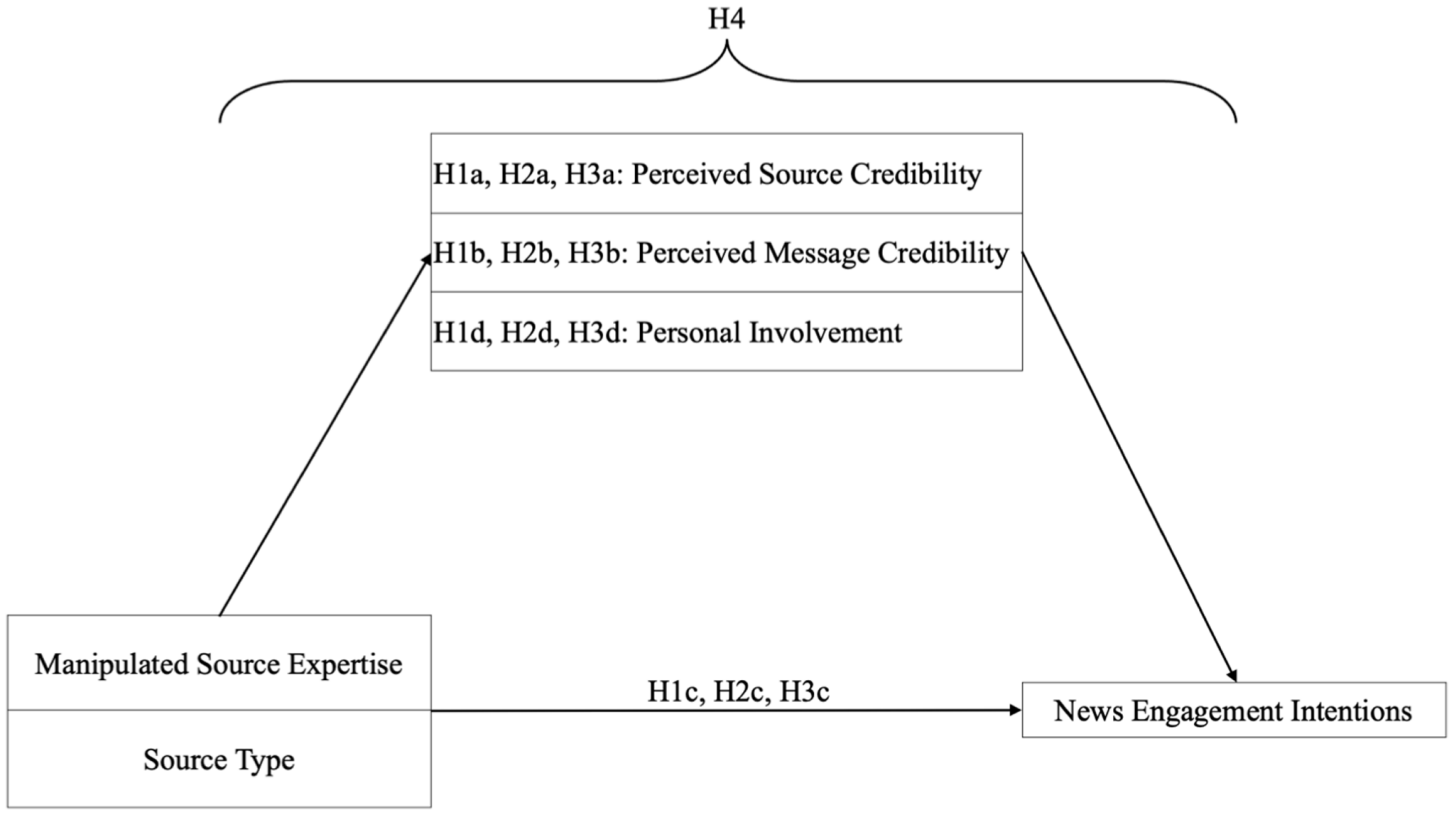
Overview of the proposed research model and hypotheses.

Since we do not manipulate the mediators experimentally and since a specific order of a causal effect cannot be derived from current theory and empirical findings, we include the mediator variables as equivalent parallel mediators rather than sequential mediators in the model, avoiding an arbitrary and guessed order, as proposed by Hayes (2012). To conclude, Figure 1 provides a graphical overview of the research model and all hypotheses of the present study.

## 3 Methods

### 3.1 Sample

We implemented an online experiment in German language with the software Unipark (Tivian, 2017). Participants were recruited via social media, flyers, and email distribution lists of various German universities. The minimum age required for participation was 18 years, and all participants gave informed consent. No personal identifying data was collected, and we offered no incentives for participation.

An *a priori* power analysis was conducted via G*Power 3.1 (Faul et al., 2009) to assess the required minimum sample size regarding the targeted 2 (manipulated source expertise: with political expertise vs. without political expertise) × 2 (source type: SNS representation of traditional news magazine vs. influencer) ANOVA based on a medium effect (*f* = 0.25), a significance level of *α* = 0.05, and a test power of 0.95. The minimum sample size was *n* = 210. A sample of 431 participants took part in this online experiment. We excluded 14 participants because they paused the experiment in the mid-term or had an implausibly long completion time, which both counteracted the experimental manipulation, and one participant was excluded as they were under the age of 18. So, the data of 416 participants were finally included in the analyses (75.2% female, 23.8% male, 1.0% diverse, *M*_age_ = 29.72, *SD*_age_ = 12.09; 75.5% having an own Instagram account). The most mentioned highest academic qualifications achieved by the participants were general matriculation standard (Abitur) (35.1%) and a bachelor’s degree (32.0%), followed by a master’s degree (25.5%), vocational training (6.7%), modern secondary school certificate (Realschule) (0.5%), and general secondary school certificate (Hauptschule) (0.2%).

### 3.2 Procedure

After providing informed consent, participants were randomly assigned to one of the following four experimental conditions by the software: (1) magazine with political expertise (*n* = 100), (2) magazine without political expertise (*n* = 102), (3) influencer with political expertise (*n* = 108), and (4) influencer without political expertise (*n* = 106). We delivered a short description of the SNS Instagram. Then, we explained that the Instagram profile “TONI” has created news posts about political topics which should now be evaluated to get a better idea of the impressions potential subscribers get of TONI and the socio-political Instagram posts. Depending on the experimental condition, TONI was introduced as either (1) a news magazine that reports daily online on current political events and keeps its followers updated on political news, (2) a fitness magazine that reports daily online on current fitness and exercise trends and keeps its followers updated on fitness news, (3) a political influencer who reports daily online on current political events and keeps their followers up to date on political news, or (4) a fitness influencer who reports daily online on current fitness and workout trends and keeps their followers up to date on fitness news. We then informed the participants that they would see the profile and the three different news posts, which were identical in all four conditions. We explained that these would be displayed for 70 s each and would proceed automatically. We also asked the participants to look at TONI’s profile and the Instagram posts as carefully as possible, as questions about TONI, the news posts, and the topic itself would be asked afterward (all instructions can be found in the Supplementary Information File).

Afterward, the Instagram profile and the three political news posts were automatically presented to the participants for 70 s each. Source credibility, message credibility, news engagement intentions, and personal involvement served as dependent variables. Accordingly, the participants gave their evaluation of the perceived profile’s (source) credibility, they rated the perceived message credibility, their news engagement intentions, and their personal involvement. Each dependent variable was assessed as a summative evaluation after all three news posts had been read.

As media trust is a strong predictor of credibility judgments for political information (Kim and Johnson, 2009) and knowledge about a topic influences the processing of content (Lucassen et al., 2013), the present study also considered participants’ prior topic-related knowledge (self-assessment) and social media trust as covariates. Finally, participants indicated their demographics and were dismissed.

### 3.3 Materials

#### 3.3.1 Profiles

**Figure 2:**
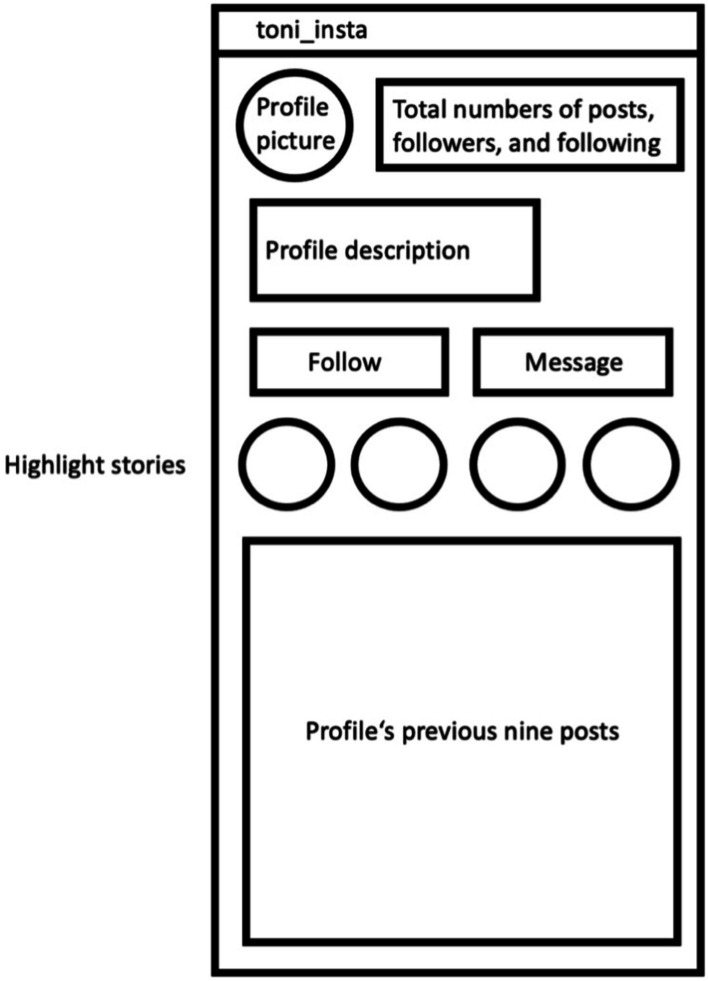
Schematic illustration of the presented Instagram profiles of the news source. The participants received one of four Instagram profiles according to the experimental condition they were assigned to. It was displayed for 70 s before showing the three Instagram news posts.

We created four different profiles of the fictitious magazine or influencer TONI, respectively, via the smartphone app Instagram. Both the magazine and the influencer had their non-working link to a respective website to increase the authenticity of their profile (*cf.* Johnson and Wiedenbeck, 2009). The profile’s look was based on existing German magazines and influencers for news and fitness topics on Instagram. They included a neutral profile picture which was the same for all four experimental conditions. Likewise, the total number of posts, followers, and following people were held constant. A plausible number within the range of a micro-influencer (up to 99 thousand followers) was chosen as these make up the largest group of social media influencers (Ruiz-Gomez, 2019). This number was same across all conditions and participants. The profiles indicated whether they had political expertise or not: First, the profile description hinted toward a focus either on politics or fitness. Second, the “highlight stories” were visible on the outline of the profile and included topics like “climate” and “national election” for the political profile, and “strength training” and “yoga” for the fitness profile. Third, the profile’s previous nine posts were shown at the bottom of the profile overview. We additionally created these previous posts which all included a headline and a corresponding image. For the political profile, the posts presented current political topics in Germany such as “Measures against COVID-19,” “The national election,” and “Digitalisation in schools.” These posts were the same for the influencer and the news magazine with political expertise. For the profiles without political expertise, we presented posts about fitness and workouts such as “Healthy nutrition,” “How to reach a perfect beach body,” and “Fitness studio alternatives.” These posts were identical for the influencer and the news magazine. We presented screenshots of these Instagram profiles to the participants. Figure 2 shows the schematic layout of these profiles. The Instagram profile thereby was the first page of a total of four Instagram pages shown. The participants received one of four Instagram profiles analog to the experimental condition they were assigned, which they viewed for 70 s. After these 70 s, they automatically moved on to the news posts.

#### 3.3.2 Targets (political news posts)

**Figure 3:**
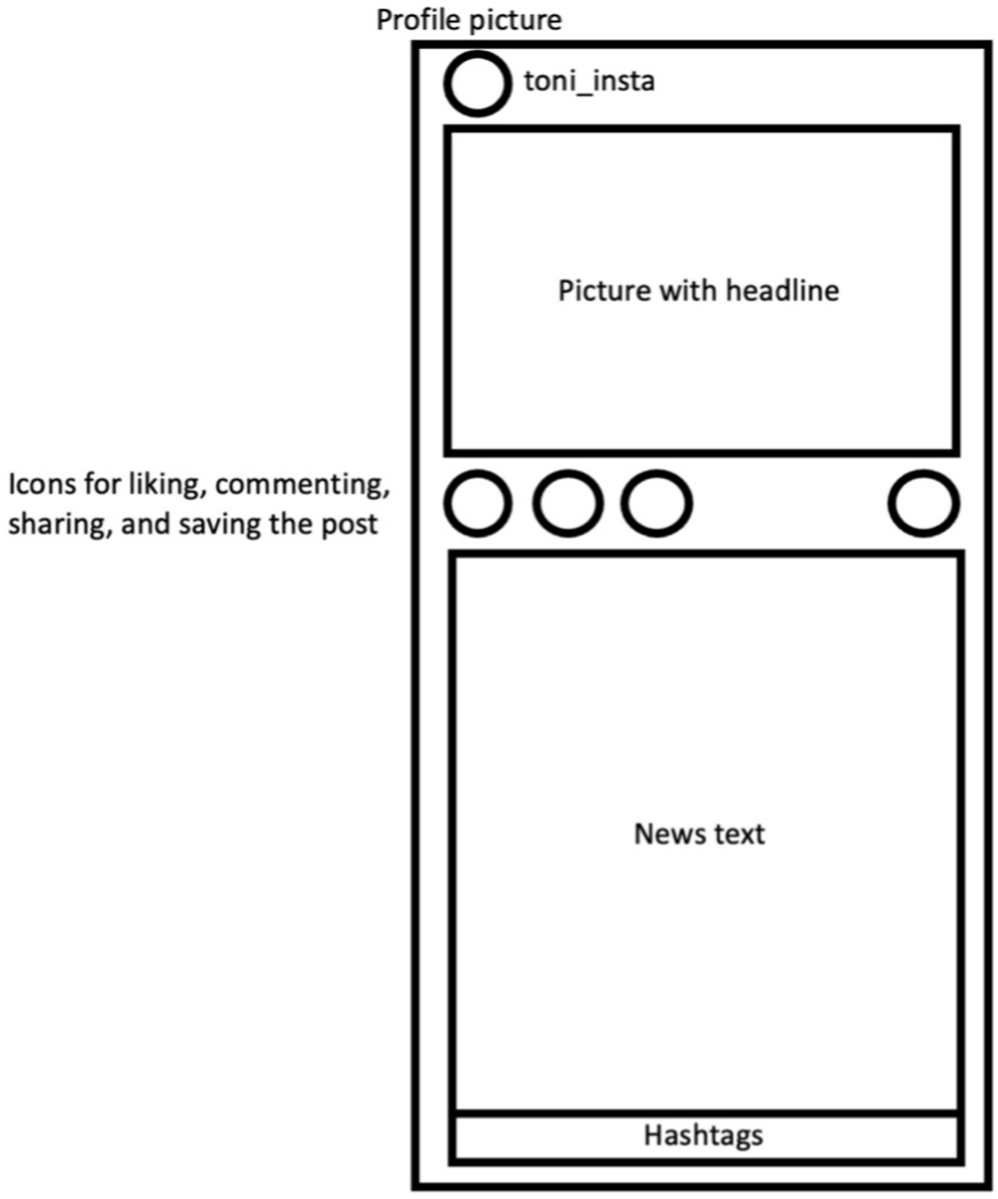
Schematic illustration of the presented Instagram posts with identical layout in all four experimental conditions.

We created a total of three news posts on socio-political topics based on actual news and news articles that were up-to-date in Germany at the time of the study. These posts included the topics of an unconditional basic income, children’s rights in the Basic Law, and unemployment benefits. The news posts each consisted of a corresponding image, a headline, the news text, and four keywords in the form of hashtags. We presented screenshots of these Instagram posts to the participants. Figure 3 shows the schematic layout of these news posts. After seeing the Instagram profile, the participants received all three news posts one after the other, which they viewed for 70 s each. The switch between the news posts was automatic.

#### 3.3.3 Pre-study

Beforehand, we tested the assumption that the profiles and news posts could be assigned as intended to the topics of politics or fitness, respectively. Therefore, nine participants from the same population, and who did not take part in the main study, rated each Instagram profile and news post on a 5-point scale (from 1 = “not at all” to 5 = “very much”) regarding the extent to which it could be assigned to eleven different categories (e.g., travel & holidays, food & cooking, politics & business, sports & fitness). The results showed that all profiles and news posts were assigned to the intended categories (*M* = 5.00, *SD* = 0 for the respective categories). In addition, the average time it took the participants in the pre-test to view the posts and the profiles was 70 s. Therefore, we chose this period as presentation time of the profiles and political news posts in the main study.

### 3.4 Measures

Detailed descriptive statistics for each of the following dependent variables and covariates, as well as the Cronbach’s α of the respective scales, can be found in Table 1.

**Table 1:** Descriptive statistics for perceived source credibility, perceived message credibility, anticipated news engagement, present news sharing intention, future news sharing intention, personal involvement, political knowledge, and social media reliance.

	Total (*N* = 416)		Magazine with political expertise (*n* = 100)		Magazine without political expertise (*n* = 102)		Influencer with political expertise (*n* = 108)		Influencer without political expertise (*n* = 106)		Cronbach’s α
	*M*	*SD*	*M*	*SD*	*M*	*SD*	*M*	*SD*	*M*	*SD*	
Perceived Source Credibility	4.89	1.21	5.18	1.12	4.42	1.15	5.40	1.02	4.56	1.26	0.93
Perceived Message Credibility	4.66	1.29	4.88	1.26	4.31	1.24	5.10	1.12	4.31	1.35	0.86
Anticipated News Engagement	2.28	0.76	2.51	0.72	2.03	0.67	2.34	0.71	2.26	0.84	0.70
Present News Sharing Intention	2.56	1.55	2.83	1.51	2.14	1.34	2.85	1.62	2.42	1.62	n.a.
Future News Sharing Intention	2.57	1.55	2.89	1.51	2.09	1.34	2.92	1.66	2.38	1.52	n.a.
Personal Involvement	4.71	1.25	5.02	1.16	4.50	1.14	4.88	1.20	4.46	1.39	0.94
Political Knowledge	4.41	1.21	4.41	1.11	4.46	1.24	4.31	1.22	4.46	2.76	0.82
Social Media Reliance	2.72	1.12	2.77	1.04	2.50	1.11	2.85	1.13	1.24	1.18	0.74

n.a., not applicable. Cronbach’s α was not calculated for Present News Sharing Intention and Future News Sharing Intention, as both are single-item scales.

#### 3.4.1 Manipulation check

To ensure that profiles with political expertise are also perceived as such, we assessed the perceived expertise using the expertise subscale of Ohanian’s (1990) Source Credibility Scale. This subscale consists of a total of five bipolar word pairs (not an expert – expert, inexperienced – experienced, unknowledgeable – knowledgeable, unqualified – qualified, unskilled – skilled) which were rated on a 7-point scale ranging from 1 to 7 (Cronbach’s *α* = 0.926). We computed the arithmetic mean score across the five ratings to obtain a single score to be used in the analyses.

#### 3.4.2 Perceived source credibility

We measured perceived source credibility of the Instagram profiles in terms of perceived trustworthiness, as it is one of the underlying concepts of source credibility besides of expertise (Ohanian, 1990; Ismagilova et al., 2020). We used the trustworthiness subscale of the Source Credibility Scale by Ohanian (1990). This subscale consists of five bipolar word pairs (undependable – dependable, dishonest – honest, unreliable – reliable, insincere – sincere, untrustworthy – trustworthy) which were rated on a 7-point scale ranging from 1 to 7. The arithmetic mean across the five scores was calculated as a single score to be included in the analyses.

#### 3.4.3 Perceived message credibility

To measure consumers’ perception of message credibility, we adapted the message credibility scale created by Appelman and Sundar (2016). Participants indicated how well the adjectives “accurate,” “authentic,” and “believable” describe the political news posts they just read. All items were rated on a 7-point scale ranging from 1 (“describes it very poorly“) to 7 (“describes it very well“).We computed the arithmetic mean score across the three ratings to obtain a single score to be used in the analyses.

#### 3.4.4 News engagement intentions

We operationalized participants’ intention to engage with news by means of three different facets: anticipated news engagement, present news sharing intention, and sharing intention of future news from TONI.

To assess anticipated news engagement, we adapted the anticipated news engagement scale by Scacco and Muddiman (2020). Participants indicated how much they agreed with the statements that they could imagine reading the articles, giving the articles a ‘like’ on Instagram, leaving a comment in the comment section, talking to someone about the articles, and paying a small fee for the full article (i.e., “I could imagine leaving a comment in the comment section of the posts”). All five items were rated on a 5-point scale ranging from 1 (“do not agree at all”) to 5 (“agree very much”). We calculated the mean score across the five ratings which we then used in the analyses.

To assess participants’ present news sharing intention, we adopted a single item by Bobkowski (2015). We asked participants “how likely would it be for you to share the Instagram posts you just saw from TONI on the topic of politics with others via social media.” Participants indicated the likelihood on a 7-point scale ranging from 1 (“very unlikely”) to 7 (“very likely”).

To assess participants’ future news sharing intention, another single item was created based on Lee and Ma (2012). We asked participants “how likely would it be for you to share future Instagram by TONI on the topic of politics with others via social media.” Participants indicated the likelihood on a 7-point scale ranging from 1 (“very unlikely”) to 7 (“very likely”).

#### 3.4.5 Personal involvement

To measure participants’ involvement with the news posts, we used the revision of the personal involvement scale by Zaichkowsky (1994). Participants rated the news posts by means of ten bipolar adjective pairs (unimportant – important, boring – interesting, relevant – irrelevant, exciting – unexciting, means nothing – means a lot to me, unappealing – appealing, mundane – fascinating, worthless – valuable, uninvolving – involving, not needed – needed) on a 7-point scale ranging from 1 to 7. The mean score across these items served as dependent variables in the analyses.

#### 3.4.6 Covariates

We captured self-assessed prior knowledge about politics by using a modified version of the subjective knowledge scale by Flynn and Goldsmith (1999). Participants indicated how much they agreed with the following statements: “I know pretty much about politics”; “I do not feel very knowledgeable about politics”; “Among my circle of friends, I’m one of the ‘experts’ on politics”; and “When it comes to politics, I really do not know a lot.” All items were rated on a 7-point scale ranging from 1 (“do not agree at all”) to 7 (“agree very much”). The arithmetic mean score across the four items was used as single score in the analyses.

In addition, we assessed participants’ general reliance on social media for political use. Based on previous studies on credibility on social media and surveys about political information-seeking behavior (Johnson and Kaye, 2014, 2016; Sterrett et al., 2019; Hölig et al., 2020), we asked the participants if they agree with the following two statements: “I often read political news on social media, e.g., on Facebook, Instagram, etc.” and “I rely on the political news that I find on social media.” Both items were rated on a 5-point Likert-like scale ranging from 1 (“do not agree at all”) to 5 (“agree very much”). We computed the mean score across the two items and used it as dependent variable in the analyses.

### 3.5 Analyses

We ran all analyses using SPSS 28. All analyses were conducted with and without the covariates of political knowledge and social media reliance. Due to methodological reasons like the danger of Type 1 error inflation (Wang et al., 2017) and inadequate modeling when testing for interactions (Yzerbyt et al., 2004), and as covariates are not part of the main model we postulated, we focus on reporting and discussing the findings of the analyses without covariates. For consistency, the results of the main analyses (ANCOVAs and tests for indirect effects) with covariates can be found in the Supplementary Tables S2, S3. Importantly, adding the covariates did not change any of the main results reported in the next sections.

To perform the manipulation check, we calculated a *t*-test for independent samples with manipulated source expertise as the independent variable and perceived expertise as the dependent variable.

We performed 2 × 2 ANOVAs with the manipulated source expertise and source type as independent variables to test H1, H2, and H3 for each of the dependent variables: perceived source credibility, perceived message credibility, news engagement intentions (i.e., anticipated news engagement, present news sharing intention, and future news sharing intention), and personal involvement. Given multiple testing, significance level was set to *p* = 0.008 (Bonferroni correction). In case of a significant interaction effect, we scrutinized simple main effects via *t*-tests for independent samples.

In the event of a significant main effect of manipulated source expertise/source type on news engagement intensions (anticipated news engagement, present news sharing intention, and future news sharing intention), we examined whether these effects are indirect effects via perceived source credibility, perceived message credibility, and the personal involvement (H4). For this purpose, we performed tests for indirect effects using Model 4 of the PROCESS v4.0 macro by Hayes (2017), with perceived source credibility, perceived message credibility, and personal involvement as three parallel mediator variables (bootstrapping method with 10.000 samples). We considered the indirect effects as significant when the 95% confidence interval did not include zero.

## 4 Results

### 4.1 Manipulation check

The *t*-test revealed a significant difference in the perceived expertise between the profile with versus without manipulated political expertise, *t*(414) = 9.86, *p* < 0.001, *d* = 0.97. As intended, the participants rated the perceived expertise of the profile with manipulated political expertise (*M* = 4.81, *SD* = 1.18) higher than that of the profile without political expertise (*M* = 3.64, *SD* = 1.23).

### 4.2 Effects of manipulated source expertise and source type (H1, H2, H3)

For each dependent variable, we calculated 2 × 2 ANOVAs with the manipulated source expertise and source type as independent variables. Perceived source credibility, perceived message credibility, anticipated news engagement, present news sharing intention, future news sharing intention, and personal involvement served as dependent variable. Table 2 shows the detailed results.

**Table 2:** Results of the 2 (manipulated source expertise) × 2 (source type) ANOVAs on all dependent variables.

DV	Manipulated source expertise			Source type			Manipulated source expertise × Source type		
	*F*	*p*	η_p_^2^	*F*	*p*	η_p_^2^	*F*	*p*	η_p_^2^
Perceived Source Credibility	51.54^***^	< 0.001	0.111	2.54	0.112	0.006	0.12	0.725	< 0.001
Perceived Message Credibility	31.27^***^	< 0.001	0.071	0.81	0.369	0.002	0.84	0.359	0.002
Anticipated News Engagement	14.83^***^	< 0.001	0.035	0.22	0.641	0.001	7.82^**^	0.005	0.019
Present News Sharing Intention	13.96^***^	< 0.001	0.033	1.06	0.303	0.003	0.78	0.376	0.002
Future News Sharing Intention	20.41^***^	< 0.001	0.047	1.13	0.288	0.003	0.78	0.377	0.002
Personal Involvement	15.43^***^	< 0.001	0.036	0.58	0.449	0.001	0.17	0.679	< 0.001

DV, dependent variable. ^*^p < 0.05, ^**^p < 0.01, ^***^p < 0.001.

We found main effects of the manipulated source expertise on all dependent variables, all *F*s(1, 412) ≥ 13.96, all *p*s < 0.001, all η_p_^2^ ≥ 0.033, supporting H1. The presence of political expertise led to a higher perception of source credibility, perceived message credibility, anticipated news engagement, present news sharing intention, future news sharing intention, and personal involvement. In contrast, there were no significant effects of the source type on any of the dependent variables, all *F*s(1, 412) ≤ 2.54, all *p*s ≥ 0.112, all η_p_^2^ ≤ 0.006, contradicting H2. Furthermore, there was an interaction effect between manipulated source expertise and source type on the anticipated news engagement, *F*(1, 412) = 7.82, *p* = 0.005, η_p_^2^ = 0.019, partly supporting H3c. The post-hoc *t*-test revealed that manipulated source expertise had a significant effect on anticipated news engagement for the news magazine, *t*(200) = 4.92, *p* < 0.001, *d* = 0.69, but not for the influencer, *t*(212) = 0.72, *p* = 0.472, *d* = 0.10. While there was no difference between the influencer with versus without political expertise, the anticipated news engagement was higher for the magazine with political expertise than for the magazine without political expertise (see Table 1). There were no other significant interaction effects, all *F*s(1, 412) ≤ 0.84, all *p*s ≥ 0.359, all η_p_^2^ ≤ 0.002, contradicting H3a, H3b, and H3d.

### 4.3 Tests for direct and indirect effects of manipulated source expertise (H4)

As there was a significant main effect of manipulated source expertise on all of the news engagement intentions, we computed mediation analyses to test for indirect effects of manipulated source expertise (dummy-coded: 0 = with political expertise, 1 = without political expertise) on each of the dependent variables “anticipated news engagement,” “present news sharing intention,” and “future news sharing intention” through the parallel mediator variables “perceived source credibility,” “perceived message credibility,” and “personal involvement.” The detailed results of the analyses can be found in the Supplementary Information File.

**Figure 4:**
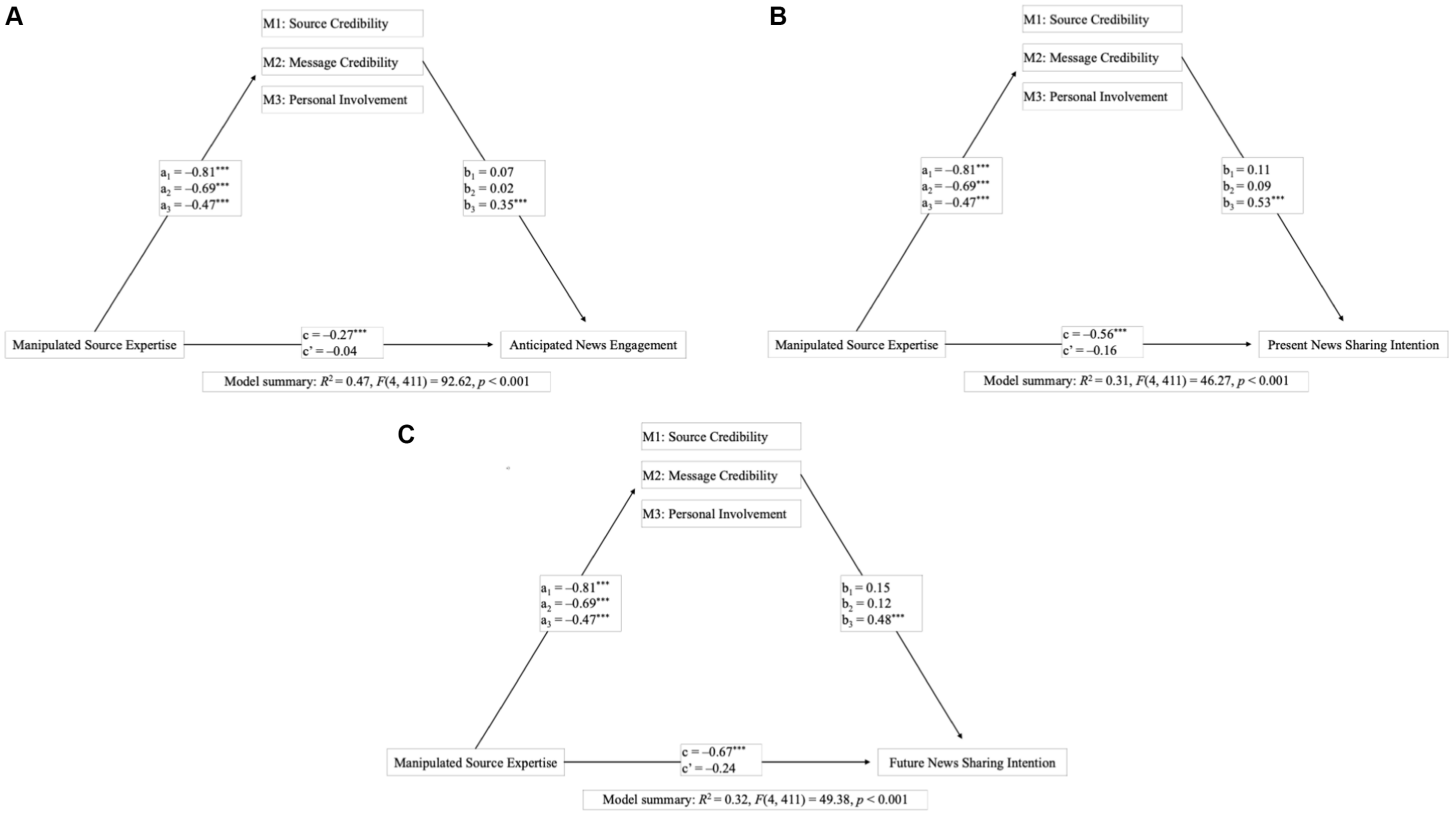
Results of the test for indirect effects of the manipulated source expertise on **(A)** anticipated news engagement, **(B)** present news sharing intention, and **(C)** future news sharing intention through perceived source credibility, perceived message credibility, and personal involvement. Manipulated source expertise was dummy-coded (0 = with political expertise; 1 = without political expertise). ^*^p < 0.05, ^**^p < 0.01, ^***^p < 0.001.

Figure 4 shows three models for the direct and indirect effects of manipulated source expertise, differing only regarding the independent variable, namely anticipated news engagement (A), present news sharing intention (B), and future news sharing intention (C). The models show the same result pattern for each of the dependent variables: First, we found significant negative total effects of the manipulated source expertise on all dependent variables, with *c* ranging from −0.27 to −0.67, all *p*s < 0.001. Second, the analyses revealed significant negative effects of the manipulated source expertise on each of the mediator variables, namely perceived source credibility, *a_1_* = −0.81, *p* < 0.001, perceived message credibility, *a_2_* = −0.69, *p* < 0.001, and personal involvement, *a_3_* = −0.47, *p* < 0.001. In turn, there were positive direct effects of the mediator variable “personal involvement” on each of the dependant variable, with *b_3_* ranging from 0.35 to 0.53, all *p*s < 0.001. There were no positive direct effects of the mediator variables “perceived source credibility,” with *b_1_* ranging from 0.07 to 0.15, all *p*s ≥ 0.051, and “perceived message credibility,” with *b_2_* ranging from 0.02 to 0.12, all *p*s ≥ 0.147. The analyses also revealed significant negative indirect effects of the manipulated source expertise on all dependent variables through “personal involvement,” with effects ranging from −0.25 to −0.17, 95%-*CI*s = [−0.40 to −0.26; −0.12 to −0.08], but not through “perceived source credibility,” with effects ranging from −0.12 to −0.06, 95%-*CI*s = [−0.26 to −0.12; 0.00 to 0.04], and “perceived message credibility,” with effects ranging from −0.08 to −0.01, 95%-*CI*s = [−0.19 to −0.06; 0.02–0.05]. At last, there were no direct effects of manipulated source expertise on any of the dependent variables when considering the indirect effects, with *c’* ranging from −0.04 to −0.24, all *p*s ≥ 0.069. These results therefore only partly support H4.

## 5 Discussion

In the context of political news on Instagram, this study investigated the potential effects of source expertise and source type on perceived message credibility, perceived source credibility, news engagement intentions, and personal involvement. In addition, we explored potential indirect effects of source expertise and source type on news engagement intentions.

### 5.1 Effects of manipulated source expertise on perceived source credibility and message credibility

We consistently found main effects of the manipulated source expertise on perceived source credibility and perceived message credibility (H1a, H1b). There were neither significant main effects of source type nor significant interaction effect between manipulated source expertise and source type on perceived source credibility and perceived message credibility (H2a, H2b, H3a, H3b). So, sources and the news they share on Instagram are perceived as more credible when source expertise is present, regardless of whether the source was the Instagram representation of more traditional news media or an influencer.

On the one hand, these results underline the importance of source expertise as a vital heuristic for assessing the credibility of a message’s source (Metzger et al., 2010; Sterrett et al., 2019; Meinert and Krämer, 2022) and as a central concept of source credibility (Ohanian, 1990). Accordingly, our results are in line with previous findings showing that the source expertise positively influences the perceived credibility of a message in online contexts (Kang et al., 2011; Yuan et al., 2019; Meinert and Krämer, 2020). The Instagram profile that appears to focus on political news and reports seems to be perceived as more credible in the context of political news. Thus, expertise is an essential precondition for credibility. However, it is often unclear how consumers recognize highly knowledgeable sources, especially on Instagram and, more broadly, on different SNS (Choi and Stvilia, 2015). This could be in a variety of ways: through previous news posts, which is also suggested by the results of the present study, their reputation, brand name, objectivity, popularity, or simply who they choose to trust (Sundar, 2008). Information on SNS is spread and discussed within seconds, so a lengthy process of verifying a person’s expertise often does not take place (Schäfer, 2020). As SNS influencers do not necessarily have political expertise (Trepte and Scherer, 2010) and their reporting is usually linked to political causes (Bause, 2021), the distribution of fake news may be facilitated. This calls for more research on heuristics that are used in credibility assessments to better understand the role of expertise in the context of SNS and the production and distribution of news.

On the other hand, our results contrast with previous findings showing traditional media and their news are still rated as more credible than SNS (Chung and Nah, 2009; Johnson and Kaye, 2016; Tandoc, 2019), as we found no significant differences between source types, namely the news magazine and the influencer. One possible explanation could be the important role of reputation in assessing the credibility of news media, especially more traditional media (Herbig and Milewicz, 1993; Brown et al., 2007; Metzger et al., 2010; Haas and Unkel, 2017). Several studies have used existing traditional newspaper names that are already reputable and well-known among people (Flanagin and Metzger, 2007; Chung et al., 2012; Chung and Nah, 2013; Johnson and Kaye, 2016). In this study, we created a fictitious news magazine and influencer, thus being unknown to the participants. Like expertise, reputation is also considered one of the cognitive heuristics for assessing credibility though (Metzger et al., 2010). Since an established reputation of the source is often missing in the SNS context (Flanagin and Metzger, 2000; Metzger, 2007), the fact that a more traditional news magazine has shared political information might not be important if the consumer is not familiar with this specific source. This suggests that especially unfamiliar sources do not differ in perceived credibility, regardless of whether the source is an influencer or the representation of a more traditional magazine. Another explanation may be the occurrence of source blindness (Pearson, 2021). Participants might not pay attention to the source and thus do not recognize the source as a more traditional news magazine or an influencer. This interpretation would be compatible with the consistent effect of manipulated source expertise that has been expressed by pictures and accompanying headlines on the Instagram profiles. Consumers mainly rely on contextual and design properties (instead of individual source characteristics) of SNS websites when evaluating the credibility of online information (Flanagin and Metzger, 2007; Pearson and Knobloch-Westerwick, 2018). Thus, the pictures could have been especially salient for the assessment of the source’s expertise and the source’s credibility in the present study. Since we manipulated the source type textually, this may have not been sufficiently salient to be considered in the evaluation of the source and the message. This assumption would be in line with the assumptions of the MAIN Model (Sundar, 2008), postulating that pictures are trusted more than textual descriptions due to the increased informative nature of visual cues, which in turn also affects the perception of credibility. This could even be more important on Instagram because of the emphasis of visual features (Vázquez-Herrero et al., 2019).

So, on the one hand, these results underline the importance of images and design features in conveying characteristics of a source. On the other hand, our findings support the need for future research about the characteristics of SNS platforms and profiles which convey important attributes like actual expertise and credibility. In this context, it is important to mention that we examined Instagram representations of traditional news media and compared them to influencers. In contrast, previous research comparing the credibility of traditional media and influencers has mainly focused on traditional media formats, including their traditional layouts, showing that traditional media are considered more credible than SNS in general (i.e., Zimmermann et al., 2020; Besalú and Pont-Sorribes, 2021). We could not find this difference for the SNS representations of traditional media. It is possible that further distinctions are made between traditional media formats and their SNS representations, leading to different consumer perceptions. One reason for this could also be layout factors, so news on SNS is only assessed as one category due to the uniform layout, regardless of whether it was shared by a SNS representation of traditional news media or an influencer. So, the examination of potential differences in perceived source credibility and message credibility between traditional news media formats and their SNS representation can be an interesting direction for future research.

Nevertheless, the results also indicate that influencers seem to be an acceptable source for political news on Instagram, as influencers are rated with the same credibility as the Instagram representation of a news magazine. In line with Xiao et al. (2018), consumers seem to accept influencers as people with knowledge and expertise. Influencers need to be considered as a new way of gathering political news and supporting their role as new gatekeepers for political news on SNS. This could lead to positive effects like an increase in political engagement as proposed by Riedl et al. (2021). Influencers often lack political focus (Bause, 2021) though, so their content might be altered (Metzger and Flanagin, 2015). Furthermore, influencers were found to rather sensationalize mainstream news and thus represent more extreme and reactionary political standpoints (Lewis, 2020), which in turn could result in the reinforcement of radical ideologies, or conspiracy theories (Riedl et al., 2021). These dangers and the growing relevance of influencers as gatekeepers indicate that opportunities for source identification and their competencies on SNS should be strengthened to avoid source blindness among consumers. In addition, approaches to educate about the credibility and potential negative impact of influencers’ political content should be developed to strengthen the ability to recognize misinformation.

### 5.2 Effects of manipulated source expertise on news engagement intentions and personal involvement

We consistently found main effects of manipulated source expertise on the three facets of news engagement intention (anticipated news engagement, present news sharing intention, and future news sharing intention) as well as on personal involvement with the news (H1c, H1d). There were no main effects of the source type on any of these variables though (H2c, H2d). Thus, the intention to engage with the news and to share the present and future political news, as well as how involved the participants felt with the news, were higher for the sources with political expertise compared to the sources without political expertise. We also found a significant interaction effect between manipulated source expertise and source type, but only regarding anticipated news engagement (H3c): there was no difference between the sources with high versus low political expertise when an influencer shared the news. However, when a news magazine shared the news, high political expertise led to significantly higher anticipated news engagement than low political expertise. This result seems somewhat surprising considering that traditional media are perceived as more credible (Johnson and Kaye, 2014) and are trusted more than SNS sources (Li and Zhang, 2018), while the expertise of influencers is often questioned (Trepte and Scherer, 2010), so thematic expertise should play a more important role for influencers. Our results may be explained by the fact that influencers are perceived as on equal terms with their followers (Bause, 2021). They act as peers to consumers and therefore often function as inspiration and advisors (Chopra et al., 2021). Therefore, they can engage their followers regardless of their expertise more easily than SNS representations of traditional news media. In contrast, the present interaction effect highlights the importance of having and conveying political expertise for SNS representations of traditional news magazines to encourage engagement with political news. The results show that professional journalists with high expertise have the chance to mobilize and motivate news consumers interacting with their news, as Nah and Chung (2012) already discussed, underlining the role of professionalism for traditional news magazines.

Importantly, the effects of manipulated source expertise on the three news engagement intention variables (anticipated news engagement, present news sharing intention, and future news sharing intention) were indirect effects mediated through personal involvement, but not mediated through perceived source credibility and perceived message credibility (H4). When considering the indirect effects, the direct effects of the manipulated source expertise on the three facets of news engagement intention disappeared. Our results thus suggest that the expertise of a news source determines how much people are involved with the news, which then may lead to news engagement intentions. We therefore propose this model to better understand how the influence of an SNS account’s expertise affects consumers’ intention to engage with that account’s political news. Expertise has a positive effect on news engagement intentions but does so only indirectly via the personal involvement of the consumers in the news. Our results once again underline the key role of personal involvement (Meinert and Krämer, 2020) for news engagement intentions, which surprisingly seems to be more important than the perceived credibility of the source and the perceived credibility of the message. This again highlights the importance of conveying one’s expertise to get consumers to interact with news on SNS. SNS representations of traditional media that indicate thematic expertise can drive consumers to their websites to read the full news article, which was shown to be a goal of traditional news outlets on SNS (Hille and Bakker, 2013). Similarly, influencers can use this knowledge to actively work with their expertise on their profiles to heighten involvement with their posted news, as well as engage, mobilize, and activate their followers. The influencer’s expertise is often a reason why a consumer follows that influencer in the first place (Chopra et al., 2021). So, influencers might have a chance to get people excited about politics who do not have much knowledge about or are not interested in it, as SNS could be an important news source for people who do not consume political news on other channels (Bode, 2016). In fact, previous research already showed that political influencers can have a positive effect on consumers’ response rate, political agenda (Curiel, 2020), and political interest (Schmuck et al., 2022). The results of the present study thereby suggest that having and conveying political expertise plays a key role in these positive effects of political influencers. Reversely, however, influencers who may be good at faking expertise and thus credibility, or who may have expertise but an extreme political position, can lead their consumers to engage with misinformation or radicalizing opinions and re-share them. Thus, an identification of the background of the source sharing a message and the developing of guidelines to do so seem to be essential to preventing (the distribution of) misinformation and radicalization.

### 5.3 Strengths and limitations

The present study has several strengths. First, the analysis included a broad sample, which is also associated with a corresponding test power with which relevant effects could be found. Second, we were one of the first to investigate the effects of source expertise and to compare Instagram representations of more traditional news media with influencers regarding political Instagram messaging in an experimental way, thus broadening the field of research. Similarly, to the best of our knowledge, the effects of source characteristics on personal involvement in the context of political SNS news have not yet been studied, so our results are completely new in this regard. Third, we focused on the platform Instagram which is the most relevant SNS platform for young people in Germany (ARD and ZDF, 2022), and which is especially relevant and popular due to its visual characteristics (Vázquez-Herrero et al., 2019). So far, the credibility of influencers and posts were mainly examined in the context of Facebook or online blogs (Yuan et al., 2019; Meinert and Krämer, 2020). Finally, we additionally investigated the mediating role of perceived source credibility, perceived message credibility, and personal involvement for the effects of source expertise on news engagement intentions, giving new indications for the relation between those variables.

In terms of limitations, the results of this study need to be considered in their social and cultural context, which can influence credibility judgments (i.e., Fogg, 2003). This study was carried out in a German setting only, which limits the generalizability of the results. Due to different values, perceptions, and habits, the results could be different in different social and cultural settings (Yang et al., 2013; Balaban and Mustățea, 2019). Second, we only examined news in the layout of the SNS Instagram which is characterized by its focus on expression and reporting through images and videos rather than text (Chen, 2018). Since it could be shown that consumers may rely on design and contextual properties more than on properties of the source (Pearson and Knobloch-Westerwick, 2018; Pearson, 2021), it could be assumed that the focus on images could be an advantage in conveying source properties such as expertise. In this context, it should also be noted that we implied the expertise of the profiles through previous posts that contained images and a headline, without the actual expertise of the source being verified by the users. How salient such properties then are on platforms that focus on text rather than images, like X (former Twitter), and how generalizable our results are across other SNS platforms remain an open question and could be a subject for future research. Third, we did not make a distinction between SNS representations of quality and tabloid newspaper. There may be a difference in the results when quality and tabloid newspapers are distinctively compared to influencers. Some influencers may be more like tabloid newspapers because of their way of presenting and editing the news. If the participants additionally perceived the SNS representation of the news magazine in this study more as a tabloid newspaper, this could explain the lack of effect of the source type. However, this is only speculative and should be further investigated in future research. Nevertheless, it should also be noted that a so-called tabloidization of news in SNS could be observed. Thus, quality newspapers online are also adopting more and more characteristics of tabloids in the presentation style of their news (Otto et al., 2017), so that this distinction blurs on SNSs. This trend would make an explicit distinction unnecessary and underlines again the importance of source expertise as heuristic for the evaluation of the source. Fourth, political information is often consumed passively as a secondary outcome of the use of the SNS feed (Bode, 2016; Schäfer, 2020), thus news is often consumed incidentally and quickly (Bergström & Belfrage, 2018; Keib et al., 2021). In the present study, we directly instructed the participants to look at the source profile and news posts thoroughly and gave them enough time to do so. In a field situation, this would not necessarily be the case when consuming the news feed. Thus, only the news post may be consumed incidentally and briefly while scrolling through the news feed, and the profile of the source is not displayed. This makes it difficult to identify the source and their expertise, so the results could change in terms of the impact of the source’s expertise. So how this plays out under field conditions remains to be seen and would be an exciting question for future research. In this context, it would also be interesting to examine actual behavior. In the present study, we were able to assess news engagement intentions due to the nature of the study. How this relates to actual news engagement remains open. Finally, we emphasize that we investigated and found indirect effects of expertise on news engagement intentions via personal involvement. Therefore, it must be noted that our proposed model is based on the examination of indirect effects and not on the study of actual (causal) mediation. Investigating causal mediation processes with an experimental manipulation of source credibility, message credibility, and personal involvement may therefore be a prospect of future research to extend our proposed model. A lack of experimental manipulation of the mediators as well as empirical evidence for the order of a causal relationship between them was also the reason why we tested the mediators in the model only as parallel mediators (Hayes, 2012). However, sequential and hierarchical mediation is conceivable and may be prospects of future research. Nevertheless, our model already provides a first basis for understanding the effect of source expertise on news engagement intentions on SNS, and the role personal involvement plays in this process.

### 5.4 General conclusion

All in all, the results of the present study contribute to our understanding of the impact of source characteristics, namely source type and source expertise, on credibility perceptions, personal involvement, and news engagement intentions in the context of political news distributed via Instagram. The present study suggests that influencers are perceived as credible as Instagram representations of more traditional news magazines. Furthermore, the different types of news sources do not seem to differently affect personal engagement or behavioral intentions. This could be since news providers are mainly judged by their reputation (Metzger et al., 2010), which was not realized and examined in this study. This, in turn, suggests that consumers (sometimes) ascribe the same credibility to both magazines and influencers, especially when they are still unknown. The study thus underscores that influencers play a relevant role in online political coverage on Instagram. However, influencers belong to the citizen journalists and thus offer a different way of spreading political news. The questions about the actual credibility and expertise of influencers as well as their level of research they do before sharing news remain open and may be a prospect for future research.

Importantly, the level of political expertise causally affect credibility perceptions, involvement, and news engagement intentions. Accordingly, it is particularly important to review the political content in relation to the expertise of the reporter. In general, influencers and their function in political and policy reporting are still little researched, which could be a prospect for future research. Deuze (2019) even believes it is important to prepare everyone to be journalists and develop SNS skills, as we are all our own reporters on SNS. Against this background, guidelines for identifying credible influencers should be developed, as they represent an important new source of information alongside traditional media. Also, guidelines for critical media research and journalistic skills should be accessible and taught early on to ensure political literacy on SNS.

## Data availability statement

The raw data supporting the conclusions of this article will be made available by the authors, without undue reservation.

## Ethics statement

Ethics board approval was not required for this study on human participants in accordance with the local legislation and institutional requirements. In Germany, as stated by the German Research Association (DFG, https://www.dfg.de/foerderung/faq/geistes_sozialwissenschaften/index.html), ethics committee approval was not required for the present survey study, because the research did not pose any threats or risks to the respondents, it was not associated with high physical or emotional stress, and the respondents were informed about the objectives of the survey in advance. At the beginning of the study, participants were informed that the data of this study will be used for research purposes only and that all data are collected anonymously. Thus, no identifying information was collected. Participants who prematurely stopped the survey were not included in the analyses and all of their data were deleted from the dataset. Informed consent to participate in this study was provided by all participants via clicking a corresponding box and all participants voluntarily participated. The studies were conducted in accordance with the local legislation and institutional requirements. The participants provided their written informed consent to participate in this study.

## Author contributions

DZ: Conceptualization, Formal analysis, Methodology, Investigation, Data curation, Visualization, Writing – original draft, Writing – review & editing. AK: Conceptualization, Methodology, Investigation, Visualization, Writing – review & editing. KK: Conceptualization, Methodology, Formal analysis, Project administration, Supervision, Resources, Writing – review & editing.
