# Supplementary material for: Political news on Instagram: influencer versus traditional magazine and the role of their expertise in consumers’ credibility perceptions and news engagement
Source: Front Psychol. 2023 Dec 6;14:1257994. doi: 10.3389/fpsyg.2023.1257994 (PMC10773820; doi:10.3389/fpsyg.2023.1257994)
Supplement: Supplementary file 1 [file Data_Sheet_1.PDF]

Supplementary Information File

Political news on Instagram: Influencer versus traditional magazine and the role of their expertise in consumers' credibility perceptions and news engagement

Daniel Zimmermann[1*], Asina Klee[1], Kai Kaspar[1]

*** Correspondence:** Daniel Zimmermann: d.zimmermann@uni-koeln.de

1. Instruction of participants

1.1 Experimental condition “news magazine with political expertise”

We want to investigate the impact of political issues on social media. To understand the reach of political messages in modern communication networks, they will be disseminated on Instagram. Instagram is a social networking platform where images and videos can be edited and shared with one’s subscribers – so-called "followers". TONI (username: toni_insta) has now created some posts on the topic of social politics. TONI belongs to the *news magazines* that report daily online about *current political events* and keep their followers up to date on news in the *field of politics*. To get a better impression of TONI, on the next page you can see TONI’s Instagram profile, which so far is mostly known for *posts about politics and business news from Germany and around the world*. We would now like to check what impressions potential subscribers get of TONI and the political Instagram posts before they are published soon. To do this, TONI kindly provided us with the posts to be published for research purposes and deactivated the Instagram profile for the duration of the study. You will now see the profile and three different posts on social politics. These will be displayed for 70 seconds each and will run automatically. We ask you to look at the TONI profile and the Instagram posts as closely as possible during this time, as you will then be asked questions about TONI, the posts, and the topic itself. There is no right or wrong when answering the questions, it's all about your opinion and impressions. If you click on "Continue", you will now see the profile of the news magazine TONI and then TONI's Instagram posts on the topic of social politics. Please note that the pictures will run automatically, and you do not have to click "Next" on the following pages.

1.2 Experimental condition “news magazine without political expertise”

We want to investigate the impact of political issues on social media. To understand the reach of political messages in modern communication networks, they will be disseminated on Instagram. Instagram is a social networking platform where images and videos can be edited and shared with one’s subscribers – so-called "followers". TONI (username: toni_insta) has now created some posts on the topic of social politics. TONI belongs to the *fitness magazines* that report daily online about *current fitness and workout trends* and keep their followers up to date on news in the *field of fitness.* To get a better impression of TONI, on the next page you can see TONI’s Instagram profile, which so far is mostly known for *posts about fitness, motivation, and daily workouts*. We would now like to check what impressions potential subscribers get of TONI and the political Instagram posts before they are published soon. To do this, TONI kindly provided us with the posts to be published for research purposes and deactivated the Instagram profile for the duration of the study. You will now see the profile and three different posts on social politics. These will be displayed for 70 seconds each and will run automatically. We ask you to look at the TONI profile and the Instagram posts as closely as possible during this time, as you will then be asked questions about TONI, the posts, and the topic itself. There is no right or wrong when answering the questions, it's all about your opinion and impressions. If you click on "Continue", you will now see the profile of the news magazine TONI and then TONI's Instagram posts on the topic of social politics. Please note that the pictures will run automatically, and you do not have to click "Next" on the following pages.

1.3 Experimental condition “influencer with political expertise”

We want to investigate the impact of political issues on social media. To understand the reach of political messages in modern communication networks, they will be disseminated on Instagram. Instagram is a social networking platform where images and videos can be edited and shared with one’s subscribers – so-called "followers". TONI (username: toni_insta) has now created some posts on the topic of social politics. TONI belongs to the group of *influencers - i.e., people with a strong presence in social media who have a certain amount of reach due to their number of followers*. In particular, TONI belongs to the group of *political influencers* who report daily online about *current political events* and keep their followers up to date on news in the *field of politics*. To get a better impression of TONI, on the next page you can see TONI’s Instagram profile, which so far is mostly known for *posts about politics and business news from Germany and around the world*. We would now like to check what impressions potential subscribers get of TONI and the political Instagram posts before they are published soon. To do this, TONI kindly provided us with the posts to be published for research purposes and deactivated the Instagram profile for the duration of the study. You will now see the profile and three different posts on social politics. These will be displayed for 70 seconds each and will run automatically. We ask you to look at the TONI profile and the Instagram posts as closely as possible during this time, as you will then be asked questions about TONI, the posts, and the topic itself. There is no right or wrong when answering the questions, it's all about your opinion and impressions. If you click on "Continue", you will now see the profile of the news magazine TONI and then TONI's Instagram posts on the topic of social politics. Please note that the pictures will run automatically, and you do not have to click "Next" on the following pages.

1.4 Experimental condition “influencer without political expertise”

We want to investigate the impact of political issues on social media. To understand the reach of political messages in modern communication networks, they will be disseminated on Instagram. Instagram is a social networking platform where images and videos can be edited and shared with one’s subscribers – so-called "followers". TONI (username: toni_insta) has now created some posts on the topic of social politics. TONI belongs to the group of *influencers - i.e., people with a strong presence in social media who have a certain amount of reach due to their number of followers*. In particular, TONI belongs to the group of *fitness influencers* who report daily online about *current fitness and workout trends* and keep their followers up to date on news in the *field of fitness*. To get a better impression of TONI, on the next page you can see TONI’s Instagram profile, which so far is mostly known for *posts about fitness, motivation, and daily workouts*. We would now like to check what impressions potential subscribers get of TONI and the political Instagram posts before they are published soon. To do this, TONI kindly provided us with the posts to be published for research purposes and deactivated the Instagram profile for the duration of the study. You will now see the profile and three different posts on social politics. These will be displayed for 70 seconds each and will run automatically. We ask you to look at the TONI profile and the Instagram posts as closely as possible during this time, as you will then be asked questions about TONI, the posts, and the topic itself. There is no right or wrong when answering the questions, it's all about your opinion and impressions. If you click on "Continue", you will now see the profile of the news magazine TONI and then TONI's Instagram posts on the topic of social politics. Please note that the pictures will run automatically, and you do not have to click "Next" on the following pages.

Supplementary Table 1: Detailed results of the tests for indirect effects of manipulated source expertise on anticipated news engagement, present news sharing intention, and future news sharing intention through perceived source credibility, perceived message credibility, and personal involvement.

**Mediator**	**Perceived Source Credibility**				**Perceived Message Credibility**				**Personal Involvement**			
**Pathway**	*Coeff.*	*SE*	*t*	*p*	*Coeff.*	*SE*	*t*	*p*	*Coeff.*	*SE*	*t*	*p*
Effect of IV on mediator	–0.81***	0.11	–7.20	< 0.001	–0.69***	0.12	–5.63	< 0.001	–0.47***	0.12	–3.92	< 0.001
**Anticipated News Engagement**												
Direct effect of mediator on DV	0.07	0.04	1.96	0.051	0.02	0.04	0.45	0.652	0.35***	0.03	11.78	< 0.001
Total effect of IV on DV; Model summary for total effect	effect = –0.27 (*SE* = 0.07), *t* = –3.75, *p* < 0.001; R^2 = 0.03, *F*(1, 414) = 14.06, *p* < 0.001											
Direct effect of IV on DV	effect = –0.04 (*SE* = 0.06), *t* = –0.67, *p* = 0.506											
Indirect effect of IV on DV through mediator	effect = –0.06 (*SE* = 0.03), *95%CI*: –0.12 – 0.00				effect = –0.01 (*SE* = 0.02), *95%CI*: –0.06 – 0.04				effect = –0.17 (*SE* = 0.04), *95%CI*: –0.26 to –0.08			
Model Summary for DV	R^2 = 0.47, *F*(4, 411) = 92.62, *p* < 0.001											
**Present News Sharing Intention**												
Direct effect of mediator on DV	0.11	0.09	1.29	0.197	0.09	0.08	1.05	0.296	0.53***	0.07	7.56	< 0.001
Total effect of IV on DV; Model summary for total effect	effect = –0.56 (*SE* = 0.15), *t* = –3.72, *p* < 0.001; R^2 = 0.03, *F*(1, 414) = 13.86, *p* < 0.001											
Direct effect of IV on DV	effect = –0.16 (*SE* = 0.13), *t* = –1.17, *p* = 0.244											
Indirect effect of IV on DV through mediator	effect = –0.09 (*SE* = 0.07), *95%CI*: –0.22 – 0.04				effect = –0.06 (*SE* = 0.05), *95%CI*: –0.17 – 0.05				effect = –0.25 (*SE* = 0.07), *95%CI*: –0.40 to –0.12			
Model Summary for DV	R^2 = 0.31, *F*(4, 411) = 46.27, *p* < 0.001											
**Future News Sharing Intention**												
Direct effect of mediator on DV	0.15	0.08	1.74	0.083	0.12	0.08	1.45	0.147	0.48***	0.07	6.88	< 0.001
Total effect of IV on DV; Model summary for total effect	effect = –0.67 (*SE* = 0.15), *t* = –4.50, *p* < .001; R^2 = 0.05, *F*(1, 414) = 20.29, *p* < 0.001											
Direct effect of IV on DV	effect = –0.24 (*SE* = 0.13), *t* = –1.82, *p* = 0.069											
Indirect effect of IV on DV through mediator	effect = –0.12 (*SE* = 0.07), *95%CI*: –0.26 to 0.01				effect = –0.08 (*SE* = 0.05), *95%CI*: –0.19 – 0.02				effect = –0.23 (*SE* = 0.06), *95%CI*: –0.36 to –0.11			
Model Summary for DV	R^2 = 0.32, *F*(4, 411) = 49.38, *p* < 0.001											

Note. Coeff. = Coefficient, SE = standard error, IV = independent variable, DV = dependent variable, CI = confidence interval.
$^{*}p$ < 0.05, $^{**}p$ < 0.01, $^{***}p$ < 0.001.

Supplementary Table 2: Results of the 2 (manipulated source expertise) × 2 (source type) ANCOVAs on all dependent variables when considering political knowledge and social media reliance as covariates.

**DV**	**Manipulated Source expertise**			**Source type**			**Manipulated Source expertise × Source type**		
	F	p	η_p^2	F	p	η_p^2	F	p	η_p^2
Perceived Source Credibility	47.35^{***}	< 0.001	0.104	1.54	0.216	0.004	0.25	0.617	0.001
Perceived Message Credibility	27.93^{***}	< 0.001	0.064	0.30	0.587	0.001	1.21	.273	0.003
News Engagement Intentions									
Anticipated News Engagement	11.94^{***}	< 0.001	0.028	0.01	0.928	< 0.001	7.15^{**}	0.008	0.017
Present News Sharing Intention	11.24^{***}	< 0.001	0.027	0.31	0.579	0.001	0.45	0.505	0.001
Future News Sharing Intention	17.19^{***}	< 0.001	0.040	0.35	0.557	0.001	0.47	0.494	0.001
Personal Involvement	12.17^{***}	< 0.001	0.029	1.88	0.171	0.005	0.41	0.839	< 0.001

Note. DV = dependent variable.
$^{*}p < 0.05$, $^{**}p < 0.01$, $^{***}p < 0.001$.

Supplementary Table 3. Detailed results of the tests for indirect effects of manipulated source expertise on anticipated news engagement, present news sharing intention, and future news sharing intention through perceived source credibility, perceived message credibility, and personal involvement when considering the covariates political knowledge and social media reliance.

**Mediator**	**Perceived Source Credibility**				**Perceived Message Credibility**				**Personal Involvement**			
**Pathway**	*Coeff.*	*SE*	*t*	*p*	*Coeff.*	*SE*	*t*	*p*	*Coeff.*	*SE*	*t*	*p*
Effect of IV on mediator	–0.76***	0.11	–6.90	< 0.001	–0.64***	0.12	–5.33	< 0.001	–0.40***	0.11	–3.48	< 0.001
**Anticipated News Engagement**												
Direct effect of mediator on DV	0.07	0.04	1.98	0.050	0.01	0.03	0.40	0.692	0.33***	0.03	10.92	< 0.001
Total effect of IV on DV; Model summary for total effect	effect = –0.23 (*SE* = 0.07), t = –3.35, p < 0.001; R^2 = 0.15, $F(3, 412)$ = 23.59, p < 0.001											
Direct effect of IV on DV	effect = –0.04 (*SE* = 0.06), t = –0.65, p = 0.516											
Indirect effect of IV on DV through mediator	effect = –0.05 (*SE* = 0.03), *95%CI*: –0.11 to –0.01				effect = –0.01 (*SE* = 0.02), *95%CI*: –0.05 – 0.03				effect = –0.13 (*SE* = 0.04), *95%CI*: –0.21 to –0.05			
Model Summary for DV	R^2 = 0.50, $F(6, 409)$ = 66.97, p < 0.001											
**Present News Sharing Intention**												
Direct effect of mediator on DV	0.11	0.08	1.27	0.205	0.08	0.08	1.03	0.306	0.48***	0.07	6.72	< 0.001
Total effect of IV on DV; Model summary for total effect	effect = –0.48 (*SE* = 0.14), t = –3.34, p < 0.001; R^2 = 0.13, $F(3, 412)$ = 20.00, p < 0.001											
Direct effect of IV on DV	effect = –0.15 (*SE* = 0.13), t = –1.15, p = 0.252											
Indirect effect of IV on DV through mediator	effect = –0.08 (*SE* = 0.06), *95%CI*: –0.21 – 0.04				effect = –0.05 (*SE* = 0.05), *95%CI*: –0.16 – 0.04				effect = –0.19 (*SE* = 0.06), *95%CI*: –0.32 to –0.08			
Model Summary for DV	R^2 = 0.34, $F(6, 409)$ = 34.33, p < 0.001											
**Future News Sharing Intention**												
Direct effect of mediator on DV	0.14	0.08	1.67	.095	0.12	0.08	1.47	0.143	0.43***	0.07	6.03	< 0.001
Total effect of IV on DV; Model summary for total effect	effect = –0.59 (*SE* = 0.14), t = –4.13, p < 0.001; R^2 = 0.14, $F(3, 412)$ = 21.51, p < 0.001											
Direct effect of IV on DV	effect = –0.24 (*SE* = 0.13), t = –1.80, p = 0.073											
Indirect effect of IV on DV through mediator	effect = –0.11 (*SE* = 0.06), *95%CI*: –0.23 – 0.01				effect = –0.08 (*SE* = 0.05), *95%CI*: –0.18 – 0.02				effect = –0.17 (*SE* = 0.05), *95%CI*: –0.28 to –0.17			
Model Summary for DV	R^2 = 0.32, $F(4, 411)$ = 49.38, p < 0.001											

Note. Coeff. = Coefficient, SE = standard error, IV = independent variable, DV = dependent variable, CI = confidence interval.
$^{*}p$ < 0.05, $^{**}p$ < 0.01, $^{***}p$ < 0.001.
